# Malacological and Parasitological Surveys on Ethiopian Rift Valley Lakes: Implications for Control and Elimination of Snail-Borne Diseases

**DOI:** 10.3390/ijerph19010142

**Published:** 2021-12-23

**Authors:** Beekam Kebede Olkeba, Pieter Boets, Seid Tiku Mereta, Belayhun Mandefro, Gemechu Debesa, Mahmud Ahmednur, Argaw Ambelu, Wolyu Korma, Peter L. M. Goethals

**Affiliations:** 1 Department of Animal Sciences and Aquatic Ecology, Ghent University, Coupure Links 653, Building F, 9000 Ghent, Belgium; pieter.boets@oost-vlaanderen.be (P.B.); Peter.Goethals@ugent.be (P.L.M.G.); 2 Department of Environmental Health Science and Technology, Jimma University, Jimma 378, Ethiopia; seidtiku@yahoo.com (S.T.M.); mahmudahmednur@gmail.com (M.A.); aambelu@yahoo.com (A.A.); wolyukorma53@gmail.com (W.K.); 3 Department of Environmental Health Science, Hawassa University, Hawassa 1560, Ethiopia; 4 Provincial Centre of Environmental Research, Godshuizenlaan 95, 9000 Ghent, Belgium; 5 Department of Biology, College of Natural and Computational Sciences, Dilla University, Dilla 419, Ethiopia; belayhunmandefro@gmail.com; 6 Department of Geography and Environmental Studies, Jimma University, Jimma 378, Ethiopia; gemechudebesa@gmail.com

**Keywords:** freshwater snails, cercarial infection, *Schistosoma mansoni*, fishermen, Ethiopian Rift Valley lakes

## Abstract

Schistosomiasis is one of the snail-borne diseases responsible for the second-highest burden of diseases among neglected tropical diseases. The use of mass drug administration to the populations most at risk is a backbone of the strategy to prevent and control schistosomiasis transmission. However, it offers no protection against re-infection, and humans are often re-exposed when they return to water bodies where snails release cercariae. Surveys on cercarial infection in snails could provide better insights on human disease risk. Hence, in this study, we investigated cercarial infection in snails and also determined the epidemiology of *Schistosoma mansoni* among fishermen at Ethiopian Rift Valley lakes. Freshwater snails were collected from the shorelines of Ethiopian Rift Valley lakes for examination of cercarial infection during 2020. Environmental data on water quality variables and physical characteristics of snail habitats were collected. Stool samples were collected from fishermen and the Kato-Katz technique was applied for the quantification of *Schistosoma mansoni* eggs. A malacological survey indicated that six morphologically distinguishable types of cercariae were found in snails. Infected snails with cercaria were more likely present in habitats with high five-day biological oxygen demand and low dissolved oxygen. The overall prevalence of *Schistosoma mansoni* infection among the fishermen at Ethiopian Rift Valley lakes was found to be 21.5%. This indicates that fishermen at Ethiopian Rift Valley lakes are one of the groups of people harboring schistosome cercariae which are potentially responsible for the transmission of schistosomiasis to lakeshore communities who have contact with lake water. Therefore, complementary medical treatment, public health interventions, environmental management and snail reduction are needed to control the transmission of schistosomiasis.

## 1. Introduction

Snail-borne diseases form an important share of parasitic diseases that pose risks to human health and cause major socioeconomic problems [1]. Schistosomiasis and fascioliasis are two of the most common snail-borne diseases worldwide which are widespread in many tropical and sub-tropical countries. The medical and economic burden of these diseases are often neglected which is why they are included in the list of the neglected tropical diseases (NTDs) [2]. Schistosomiasis is responsible for the second highest disease burden among NTDs [3], with almost 93% of the 230 million infected people worldwide living in African regions [4]. Fascioliasis is another important snail-borne disease that affects livestock and humans throughout the world [5]. Traditionally regarded as a disease of livestock, fascioliasis is now recognized as important emerging zoonotic disease of humans [6]. An estimated 2.4 million people are infected worldwide while 180 million people are at risk of infection [7]. Ethiopia is one of those countries with the highest number of cases of human schistosomiasis where 38.3 million people are either infected or live in schistosomiasis endemic areas [8]. Both diseases, schistosomiasis and fascioliasis, share similarities in their life cycle, of which the most prominent feature is the infection of specific freshwater snails that act as intermediate hosts [9,10,11]. Many species of freshwater snails belonging to the genera *Biomphalaria*, *Bulinus* and *Oncomelania* are intermediate hosts of different trematode parasites of medical and veterinary importance [2]. In Ethiopia, snails of the genus *Biomphalaria* (*B. pfeifferi* and *B. sudanica*) *Bulinus* (*Bu. abyssinicus* and *Bu. africanus*) are intermediate hosts of *S. mansoni* and *S. heamatobium*, respectively [12]. Snails of the genus *Lymnaea* (*L. natalensis* and *L. truncatula*) are intermediate hosts of *Fasciola* parasites [13,14].

The transmission cycle of snail-borne diseases starts when urine or feces containing parasites are deposited in freshwater bodies and the hatched miracidia infect the snail intermediate hosts [15]. In the snails, the miracidium develops into a mother sporocyst. In the schistosomes, the sporocyst develops into the second generation sporocysts, after which in the infective larvae, cercariae are formed. In some hermaphroditic trematodes (e.g., liver flukes), the mother sporocyst develops into rediae which produce cercariae [15]. Once the cercariae are released into the water, they either penetrate the skin of the definitive host (e.g., schistosomes) or are ingested after encysting as metacercariae in or on edible plants or animals. After entering the definitive host, the schistosome larvae mature into adult worms in the blood vessels of the liver, intestine and bladder. The worms lay thousands of eggs that causes damage as they grow through tissues and consequently, infection occurs accordingly [16]. The cycle perpetuates when infected human/animal defecate or urinate into freshwater sources [15]. The populations that are at risk of schistosomiasis include school-aged children and adults in endemic areas and people with occupations that put them in direct contact with potentially infectious waters, such as fishermen, farmers, irrigation workers and women fetch/transport water for domestic use [4,17].

The World Health Organization (WHO) guided schistosomiasis prevention and control strategies depending on mass drug administration (MDA) [18,19]. Similarly, following the London declaration on NTDs in 2012 [20], the Federal Ministry of Health (FMoH) of Ethiopia developed a national master plan for combating the country’s most common NTDs to attain a transmission break plan by 2025 [21]. For schistosomes, the target is to control morbidity by means of MDA of Praziquantel to the population at risk. The MDA strategies are effective in reducing morbidity associated with schistosomes by decreasing the worm burden and the intensity of infection [15]. Although MDA is the backbone to break the transmission of schistosomiasis, the prevalence of the disease remains very high in many countries [22,23]. Mass drug administration offers no protection against re-infection, and humans are often re-exposed when they return to water bodies with snail releasing cercariae [24,25]. Hence, MDA is not effective as sole component to limit the transmission of schistosomiasis in high prevalence regions [26,27]. Snail control has appeared to be a more effective strategy to reduce the transmission of snail-borne diseases in several countries [28,29,30]. However, snail control efforts that make considerable reductions in density of snail intermediate hosts at water-access points can sometimes fail if infected snails remain [31]. In recent times, it has been shown that information on cercarial infection in freshwater snail intermediate hosts could provide a better prediction of human disease risk rather than investigating snail population size alone [32,33,34].

Therefore, this study aimed to (i) investigate cercarial infection in freshwater snails and (ii) determine the epidemiology of *S. mansoni* infection among fishermen, an important group being at risk, at Ethiopian Rift Valley lakes and its associated risk factors. The findings of this study can be used to provide information on the importance of a holistic approach to support the prevention and control of schistosomiasis.

## 2. Materials and Methods

### 2.1. Study Area 

**Figure 1:**
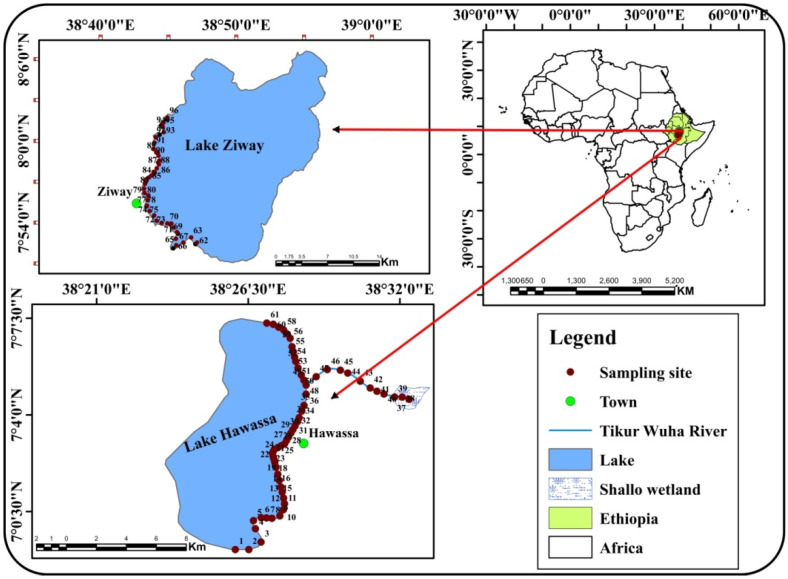
Map of the study area showing locations of sampling sites. The map was constructed using the geographic information system (GIS) software ArcGIS 10.7.

The study was conducted on Ethiopian Rift Valley lakes, namely Lake Hawassa and Lake Ziway which are situated in central Ethiopia (Figure 1). Lake Hawassa is located in the tropical rainy climate zone at a distance of 271 km from Addis Ababa, the capital city. It is situated between latitudes 06°0.97′ N and 07°0.23′ N and longitudes 38°0.37′ E and 38°0.47′ E at an elevation of 1685 m above sea level, covering a total area of km^2^, with an average depth of 11 m [35,36]. Lake Ziway is located in the warm temperate rainy climate zone at a distance of about 160 km from Addis Ababa. It has an open water area of 434 km^2^, with an average depth of 4 m. It is situated between latitudes 07°0.85′ N and 08°0.01′ N and longitudes 38°0.72′ E and 38°0.83′ E at an elevation of 1636 m above sea level [37,38]. Ethiopian Rift Valley lakes provide social, economic and ecological benefits for the local communities in the area. In spite of these benefits, there is a high probability of acquiring snail-borne diseases as there is frequent human-water contact for purposes such as domestic use, irrigation, livestock watering, fishing, recreation and alike. Fishing is an off-season activity dominant in both zones through which fishing communities get in contact with water possibly contaminated with trematode parasites.

### 2.2. Sampling Site Selection

Shorelines of the Ethiopian Rift Valley lakes and Tikur Wuha River (the tributary of Lake Hawassa) where there was evidence of human-water contact activities were selected as sampling sites for data collection. Data on environmental factors (water quality variables and snail physical habitat characteristics) and cercarial infections of freshwater snail intermediate hosts were collected from each sampling site. Data collection was carried out during the dry (March) and wet (November) seasons in 2020 at Lake Hawassa, whereas only during the wet season (November) in 2020 at Lake Ziway. During the dry season, data collection at Lake Ziway could not be carried out due to interruption by the worldwide COVID-19 pandemic. 

### 2.3. Environmental Variables 

Physico-chemical water quality variables including pH, water temperature, dissolved oxygen and electrical conductivity were measured onsite using a portable Multiprobe Meter (HQ40d Single-Input Multi-parameter Digital Meter, Hach Company, Loveland, CO, USA). Turbidity was measured onsite using a turbidity meter (Wag-WT3020; Halma PLC Company, Amersham, UK). Chlorophyll-*a* was measured onsite using a hand-held fluorometer (Aqua Fluor; Turner Designs, San Jose, CA, USA). A water sample (2000 mL) was taken from each sampling site in polyethylene bottles and transported to the laboratory using an ice cooler box for analysis of other water quality variables. In the laboratory, a water sample (250 mL) was filtered through a 45 µm filter paper and then analyzed for concentrations of total hardness and ions such as calcium, magnesium and chloride. An unfiltered water sample was used for the determination of total suspended solids, and five-day biological oxygen demand (BOD_5_). These analyses were carried out according to the standard methods for the examination of water and wastewater [39].

**Figure 2:**
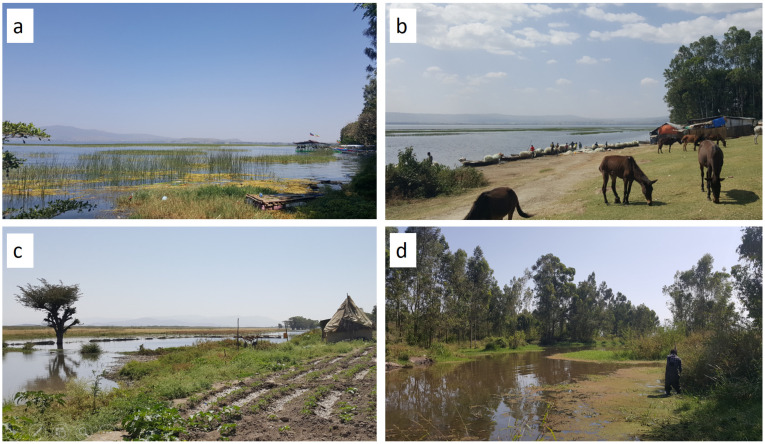
Pictures taken at sampling sites in the study area: Lake Hawassa (**a**,**b**); Lake Ziway (**c**); and Tikur Wuha River (**d**). Data were collected at Lake Hawassa and Tikur Wuha River during both the dry (15–22 March) and wet (13–30 November) seasons in 2020, but only during the wet season (13–30 November) at Lake Ziway.

The percentage of macrophyte (emergent, submerged and floating) cover was visually estimated at each sampling site [40]. The percentage of the macrophyte cover was categorized into five groups: very low (<10%); low (10–35%); moderate (35–65%); high (65–90%); and very high (>90%) [41]. Canopy cover was estimated visually based on the percentage of shade [42]. Water depth was measured using a graduated stick calibrated in centimeters. Transparency of water was determined with a Secchi disk 30 cm in diameter attached to a calibrated cord. Ambient temperature was measured using a mercury-in-glass thermometer (THL-210-050T; Vintage Gallenkamp Griffin, England). The type of substrate was carefully assessed by observation and classified into detritus, silt, sand, gravel, cobble, boulder or bedrock [43]. The presence or absence of anthropogenic activities taking place at each sampling site was recorded following direct observations (Figure 2). The common anthropogenic activities recorded were: fishing, farming/cultivation, washing/bathing, swimming/playing in the water, open defecation/urination, livestock watering and water abstraction for irrigation and industry.

A hand-held global positioning system (GPS) instrument (GPS 72H; Garmin Ltd., Olathe, KS, USA) was used to record altitude and coordinates (latitude and longitude) at each sampling site.

### 2.4. Snail Collection and Examination of Cercarial Infection 

Freshwater snails were collected using long-handheld scoops [44]. Two experienced and well-trained persons carried out scooping for 30 min. The scoop was pushed through vegetation; the biomphalarid snails were picked out of the scoops by hand using gloves and placed in plastic containers containing water and vegetation from the same habitat to transport them to the laboratory. In the laboratory, the biomphalarid snails were morphologically identified to species level using the standard identification keys [45].

The biomphalarid snail species were examined for cercarial infections by the natural shedding method following procedures used in previous studies [34,44,46]. The snails were rinsed with aerated (dechlorinated) tap water to remove the mud from their shells and placed individually in beakers containing 10 mL aerated tap water and exposed to sunlight for 1–4 h to induce shedding cercariae. The time for cercariae shedding was carefully selected to coincide with the early peak shedding time (mid-day). The water in the bottle was then checked frequently for cercariae shedding with a hand lens. If any beaker confirmed the presence of cercaria, a sample of water was transferred to slides using a dropper and stained with iodine solution and covered by a cover slip for cercariae identification. Shed cercariae were morphologically identified to genus level with a light microscope (100×) (TK-C921BEG; Victor Company of Japan Limited, T2 Tokyo, Japan) and identification keys [47]. Snails that did not shed on the first exposure were re-exposed to sunlight for the cercariae shedding every day for another consecutive seven days. During this course of time, the snails were fed lettuce in the containers containing aerated water which was replaced daily. The genus of the cercariae released by each snail was recorded. Pictures of the cercariae were taken by digital camera fitting the eye lens of the microscope (SM-G920F).

### 2.5. Mapping Spatial Distribution of Sampling Sites 

The type of land use/land cover at each sampling site in a 10 m stretch starting from the lakeshore and moving outwards was assessed [48] and then checked with the map templates of land use/land cover types of the study area computed from satellite images. The Sentinel-2 images of the study area were downloaded from the United States Geological Survey website (https://earthexplorer.usgs.gov; accessed on 25 March 2020) from which land use/land cover types were computed. Sentinel-2 images with spatial resolution of 10 m were used to assess land use/land cover of the study area through earth resource data analysis system (ERDAS) 2015 image processing software. Images used dated from the dry season of 2020. The catchment landscapes (land use/land cover types) adjacent to sampling sites were classified into five categories, including built-up, farmland, marshy land, wetland or water body based on standard guidelines [48]. A confusion matrix was employed to assess the classification accuracy. Accuracy of the classified land use/land cover maps were assessed using a combination of overall accuracy, producer’s accuracy, user’s accuracy, errors of commission and omission [49] and kappa coefficient [50]. 

Maps of the study area and each land use/land cover types were mapped and visually digitized using the satellite image in the geographic information system (GIS) software packages ERDAS 2015, ArcGIS 10.7 and validated by ground truth points.

### 2.6. Parasitological Survey and Assessment of Risk Factors 

The parasitological survey was carried out to determine the prevalence and infection intensity of *S. mansoni* among fishermen at Ethiopian Rift Valley lakes. Despite the fact that the malacological survey included all cercarial infections in biomphalarid snails, the parasitological survey focused on *S. mansoni*, a severe intestinal infection in Ethiopia. Based on the WHO monitoring guidelines [51], stool samples were collected from 200 fishermen at each Lake. Fishermen who were members of fishermen associations and had no history of taking Praziquantel (anthelmintic) in the past 6 months were considered as possible participants of the study. They were purposively selected based on their availability at the landing place.

A unique identification number was given to each participant. Each participant was provided with a labeled stool cup with an applicator stick for a stool sample after orienting them on how to provide a sufficient stool sample. Collected stool samples were processed using a Kato–Katz technique [52]. For quantification of *S. mansoni* eggs the samples were examined using a light microscope (TK-C921BEG; Victor Company of Japan Limited, T2 Tokyo, Japan). The egg of *S. mansoni* in the Kato slides was counted and multiplied by 24 to convert eggs per gram of stool (EPG). The intensity of infection was classified as light (1–99 EPG), moderate (100–399 EPG), and heavy infections (≥400 EPG) [53].

A questionnaire was used to assess the practices of the fishermen to prevent and control schistosomiasis transmission.

### 2.7. Ethical Approval and Consent to Participate

Ethical clearance was obtained from the institutional review boards (IRBs) of the Institute of Health Sciences, Jimma University. A formal letter was written to all concerned bodies and permission was secured at all levels. The objectives of the study were explained to the study participants and written consents were obtained. The confidentiality of the information was assured, and the respondent’s privacy was maintained. Participants with positive results in the microscopic examination test for *S. mansoni* were referred to the nearby health facility for treatment. 

### 2.8. Data Analysis

Data analyses were carried out using R software (Version 3.5.2) [54]. The prevalence of cercarial infection in snails was determined as a percentage, by dividing the number of snails that shed cercariae by the total number of snails examined and multiplying the outcome by 100. Shapiro–Wilk normality tests for normality and homogeneity of variance showed that data were not normally distributed. Hence, a non-parametric Kruskal–Wallis test was performed to test whether significant differences in the number of infected snails existed among physical characteristics of the snail habitats (i.e., category of macrophyte cover and substrate type). A Wilcoxon post-hoc multiple comparison test was performed to identify significantly different pairs. The post-hoc test was *Bonferroni* corrected. 

Logistic regression analysis was used to identify the factors that significantly influence the occurrence (presence/absence) of infected snails. Spearman’s rank-order correlation was used to determine associations between the number of infected snails and environmental (physico-chemical) variables. 

Prevalence and intensity of *S. mansoni* infection were reported in percent and mean egg count, respectively. Risk factors associated with *S. mansoni* infection among the fishermen were analyzed by bivariate logistic regression followed by a multiple logistic regression model. The magnitude of association was measured through odds ratio at the 95% confidence interval. *p*-values less than 0.05 were considered to be statistically significant.

## 3. Results

### 3.1. Cercarial Infection in Snails 

A total of 169 biomphalarid snails were collected from 61 sampling sites on the shorelines of Lake Hawassa during both dry and wet seasons, while 88 biomphalarid snails were collected from 35 sampling sites on the shorelines of Lake Ziway during the wet season. Overall, a total of 257 snails were collected from the total of 96 sampling sites on the shorelines of Ethiopian Rift Valley lakes, 78 snails were infected which accounted for an infection prevalence of 30.5% (pooling all cercariae identified).Infected snails were encountered at 20 sampling sites out of the total number of 96 sampling sites (20.8%). During the wet season, a higher prevalence of cercarial infection in snails (49.2%) was encountered at Lake Hawassa compared to Lake Ziway (36.7%). The prevalence of different types of cercarial infection in snails and the number of infected snails are summarized in Table 1.

**Figure 3:**
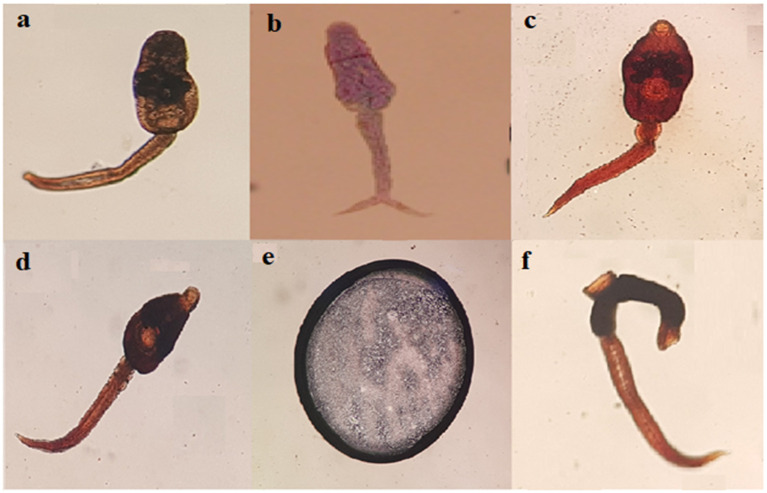
Microscopic images of different types of cercariae that shed by biomphalarid snails: (**a**) Amphistome cercariae; (**b**) BAD cercariae; (**c**) Echinostome cercariae; (**d**) Gymnocephalous cercariae; (**e**) Metacercariae; (**f**) Ornatae xiphidiocercariae. All cercariae identified to the genus level were 2 mm in size.

Collectively, six morphologically distinguishable types of cercariae were found in biomphalarid snails. These are amphistomes, brevifurcate-apharyngeate distome (BAD), echinostome, gymnocephalous, metacercariae, and ornatae xiphidiocercariae (Figure 3). 

Concurrent infections, with more than one type of cercaria, were observed in a single biomphalarid snail. With the exception of metacercariae which was only found in *B. pfeifferi*, the other five types of cercariae were found in both *B. pfeifferi* and *B. sudanica*. The highest infection prevalence was recorded for BAD cercariae in biomphalarid snails collected from Lake Hawassa (37.5%) during dry season, whereas the highest infection prevalence was recorded for echinostome cercariae (30.2%) during the wet season. Echinostomes were the most prevalent cercariae released by biomphalarid snails collected from Lake Ziway (16.6%) during the wet season. 

### 3.2. Spatial Distribution of Infected Snails

**Figure 4:**
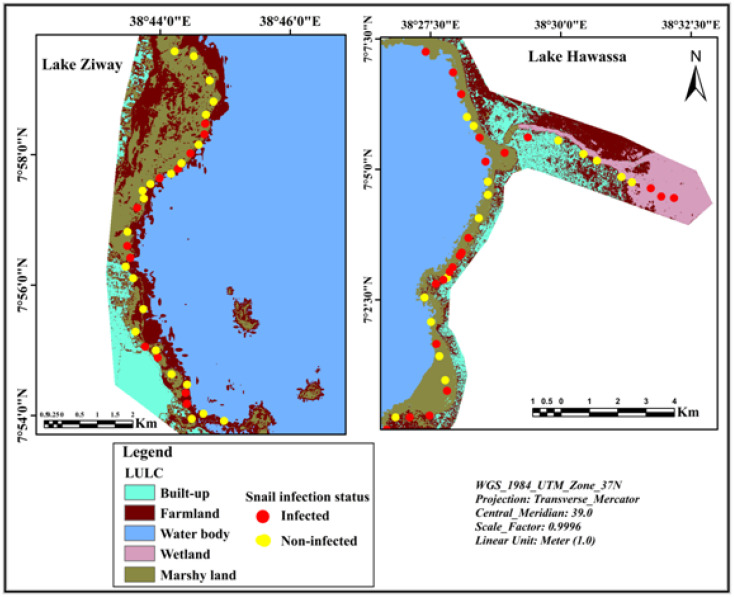
Mapping of hotspots of infected snails in relation to land use/land cover types on the shorelines of Ethiopian Rift Valley lakes.

The spatial mapping of sampling sites indicated that infected snails were distributed in habitats surrounded by all land use/land cover types found in the study area. However, the presence of infected snails was most frequently collected from sampling sites located adjacent to farmland (Figure 4).

### 3.3. Factors Affecting the Occurrence of Infected Snails

Descriptive statistics of the environmental variables determined at snail collection sites are given in Table 2. The output of the logistic regression analysis revealed that snails living in water with low dissolved oxygen and high BOD_5_ were more likely infected (Table 3). The number of infected snails was negatively associated with dissolved oxygen and water transparency, but positively associated with water turbidity (all *p* < 0.05).

### 3.4. Schistosoma Mansoni Infection and Associated Risk Factors

The result of the parasitological survey indicated that the overall prevalence of *S. mansoni* among the fishermen at Ethiopian Rift Valley lakes was found to be 21.5% (86/400). Comparing the two lakes, *S. mansoni* infection was more prevalent among the fishermen at Lake Hawassa, 31% (62/200) than those at Lake Ziway, 12% (24/200). The overall infection intensities recorded for *S. mansoni* were categorized as light, moderate and heavy among the fishermen at the Ethiopian Rift Valley lakes (Table 4). The majority of overall infections were categorized as light with EPG ranging from 24 to 2112. Specifically, the distribution of infection intensities of *S. mansoni* was fairly even among fishermen at Lake Hawassa with one-third of the fishermen having light, one-third having moderate and one-third having heavy signs of infection, whereas most of the infection intensities were categorized as light among fishermen at Lake Ziway.

Multiple logistic regression analysis indicated that the odds of infection by *S. mansoni* was significantly associated with the age of the fishermen, habit to defecate in the shorelines of lakes, using water from the lakes for domestic purposes and the type of activity to which the fishermen are engaged (Table 5). The odds of infection by *S. mansoni* were 79% less among fishermen aged between 18 and 27 years compared to fishermen aged 38 years and above (AOR = 0.21; 95% CI: 0.07–0.64). Likewise, the odds of infection among fishermen aged between 28 and 37 years were 63% less compared to fishermen aged 38 years and above (AOR = 0.37; 95% CI: 0.13–0.96).

The odds of infection by *S. mansoni* were 2.37 times higher among fishermen with the habit to defecate in the shorelines of lakes compared to the counter parts (AOR = 2.37; 95% CI: 1.37–4.16). The odds of infection by *S. mansoni* were 2.24 times higher among fishermen who engaged in fishing activity compared to those who engaged in fish processing (AOR = 2.24; 95% CI: 1.28–3.91). 

On the contrary, fishermen who used water from the lakes for domestic purposes were 67% less likely to acquire *S. mansoni* infection compared to those who did not use water from the lakes for the same purposes (AOR = 0.33; 95% CI: 0.14–0.75).

## 4. Discussion

This study is one of the few studies investigating the infection prevalence of cercariae in freshwater snails. In this study, the malacological survey revealed that six morphologically distinguishable types of cercariae are shed by biomphalarid snails which accounted to an infection prevalence of 30.5%. This finding is higher than the infection prevalence of cercariae in snails reported from Omo Gibe river basin, southwest Ethiopia (3.6%) [44], Chitwan district, central Nepal (3.5%) [55], and Kavre, Nepal (1.7%) [56]. Previous research has reported that cercarial infection in freshwater snails could be due to contamination of the water bodies by feces of human beings, aquatic birds, and domestic and/or wild animals being present in the catchment area [57,58]. 

Previous studies have demonstrated that cercarial infection in freshwater snails is associated with water quality variables and physical characteristics of snail habitats [34,44,59]. In this study, snails living in water with high BOD_5_ and low dissolved oxygen were more likely infected. In addition, the number of infected snails was positively associated with water turbidity, but negatively associated with dissolved oxygen and transparency of the water. This could be related to contamination of water with human feces and the presence of organic matter that snails feed upon. Well-fed snails tend to produce more parasites [60,61]. Consequently, humans and animals that drink lake water or come into contact with cercariae-infested water are at risk of infection. At the time of data collection in this study, infected snails were collected from lakeshores where anthropogenic activities (i.e., open defecation, washing and bathing, swimming and children playing in the water, and others) were present. Similarly, it has been documented that water pollution caused by people also promotes the occurrence of snails being infected with cercariae [44,62]. Open defecation in and around water bodies, fields, forests, bushes, or other open spaces is common practice in low-and middle-income countries [44,63,64]. These practices may result in the release of *Schistosoma* eggs into water bodies where they hatch and release miracidia, which enter into snail hosts and release cercariae [26]. Infection with cercariae occurs when humans are exposed to water bodies infested with cercariae released by snail intermediate hosts [65]. 

In this study, the presence of BAD cercariae (schistosome cercariae) in biomphalarid snails is a potentially important digenean of medical importance which could be linked to the transmission of *S. mansoni* infection among fishermen in the study area. Biomphalarid snails can serve as an intermediate hosts for the *S. mansoni* parasite in Ethiopia [66,67]. A parasitological survey carried out in this study showed that the overall prevalence of *S. mansoni* among fishermen at Ethiopian Rift Valley lakes was found to be 21.5%. This finding is higher than reports on the prevalence of *S. mansoni* among fishermen from different African countries, such as Burkina Faso (16.35%) [61] and Zambia (12.6%) [68], but lower than the prevalence reported from Ethiopia (29.2%) [69] and Egypt (26.6%) [70]. This variation might be due to the differences in environmental factors that favor the distribution of snail intermediate hosts, frequency of human-water contact, endemicity in the area, and others. Moreover, differences in personal and environmental sanitation levels might be responsible for the variation of *S. mansoni* infection from place to place. In this study, defecation on the shorelines of lakes was found to be a risk factor associated with *S. mansoni* infection among fishermen. Overall, the fishermen at Ethiopian Rift Valley lakes are one of the groups of people at risk of *S. mansoni* infection and that might be responsible for the transmission of schistosomiasis to other segments of the lakeshore community. However, fishermen using lake water for domestic purposes were less likely infected with *S. mansoni* which is probably due to the effect of household water treatment (i.e., boiling, chlorination) and/or poor report by fishermen on the practices to prevent and control schistosomiasis transmission. Unless appropriate measures are taken to protect the lakes from pollution, the range of suitable habitats for snail intermediate hosts can further extend and threaten the health of nearby residents. 

In principle, individual protection from schistosomiasis infection can be achieved by avoiding contact with water infested with schistosome cercariae. However, for people living in areas of Ethiopian Rift Valley lakes, water-human contact is often unavoidable as their daily lives are dependent on the lakes. Therefore, the Ethiopian Rift Valley lakes should be protected from disturbance by anthropogenic activities in order to control schistosomiasis in a sustainable way. For instance, there is an urgent need to establish a buffer zone to reduce pollutants entering Lake Ziway. In the case of Lake Hawassa, there is a good start to establish a buffer zone to retain the pollutants from urban runoff, but it should be expanded to other areas surrounding the lake to reduce pollutants originating from agricultural areas. 

In addition to protecting the lakes from pollution, there should be public toilets available in strategic places around the lakes so that people especially fishermen and farmers can defecate safely during occupational activities on the field as well as when they are not in their houses. There is also a need for behavioral change, because even if toilets are available, people still need to be convinced to cease open defecation. Health workers and local authorities should give health education on the health problems associated with open defecation and lake pollution. Several countries succeeded in eliminating schistosomiasis as a public health problem through integrated intervention tools [71,72]. 

Although this study has shed some light on cercarial infections in Ethiopia, it also had some limitations. This study focused only on biomphalarid snails for the examination of cercarial infection, further study is suggested to investigate cercarial infection of other freshwater snails (i.e., lymnaeid snails). The presence of BAD cercariae in snails does not constitute robust epidemiological information unless schistosome cercariae are precisely identified to species level by molecular techniques. Hence, molecular techniques are useful to differentiate human schistosome cercariae from non-human schistosomes or cryptic cercariae (such as a bird or wildlife parasites) shed by the same snail species. In addition, molecular techniques are required to determine pre-patent stages of cercarial infection of snails to improve the detection of snail infectivity. A more integrated analysis on fisheries and ecosystem management in the context of sustainability as indicated by Forio and Goethals [73] and Gebremedhin et al. [74] would be useful to tackle these and other major needs of freshwater ecosystems [75].

## 5. Conclusions

This study revealed the presence of six morphologically distinguishable types of cercariae in biomphalarid snails collected from Ethiopian Rift Valley lakes. According to a parasitological survey, fishermen and people frequently visiting the lake water are at highest risk of *S. mansoni* infection where infected snails are present in the environment. Therefore, there is a need to apply medical treatment accompanied by public health interventions, environmental management and snail control to reduce the transmission of schistosomiasis and avoid re-infection with trematode cercariae in such settings. There is also a need to promote health education to increase awareness of the fishermen and the community in the area on the practices of the prevention and control of snail-borne diseases. Furthermore, enforcement of existing environmental, fisheries and public health laws along lakeshore inhabitants is essential to tackle problems in an integrated manner.

## Figures and Tables

**Table 1:** Prevalence of cercarial infection in biomphalarid snails collected from the shorelines of Ethiopian Rift Valley lakes (BAD = Brevifurcate-apharyngeate distome cercariae, Echis = Echinostome cercariae, Xior = Ornatae xiphidiocercariae, Gymn = Gymnocephalous, Amph = Amphistome, Meta = Metacercariae).

Study Area	Season	Snail Species	Infection Prevalence with a Type of Cercaria (%)					
			BAD	Echis	Xior	Gymn	Amph	Meta
Lake Hawassa	Wet	*B. pfeifferi*	9	30	7	2	12	5
		*B. sudanica*	0	0	0	0	13	0
	Dry	*B. pfeifferi*	0	0	0	0	0	0
		*B. sudanica*	6	4	1	2	3	0
Lake Ziway	Wet	*B. pfeifferi*	4	16	4	6	12	0
		*B. sudanica*	0	14	0	0	5	0

**Table 2:** Descriptive statistics for environmental variables used to assess the occurrence of infected snails at Ethiopian Rift Valley lakes (TSS = total suspended solids, NTU = nephelometric turbidity unit, SD = standard deviation).

Environmental Variable	Unit	Mean	SD	Minimum	Maximum
pH	-	9	1	6	10
Turbidity	NTU	20	31	2	247
Dissolved oxygen	mg/L	5	3	0.5	17
Chlorophyll-*a*	µg/L	25	13	11	76
Electrical conductivity	µs/cm	564	243	71	940
BOD_5_	mg/L	26	40	0.3	184
TSS	mg/L	43	31	5.2	136
Total hardness	mg/L	68	22	24	120
Calcium ion	mg/L	49	18	16	100
Magnesium ion	mg/L	19	8	0	36
Chloride ion	mg/L	29	9	11	48
Water depth	m	0.6	0.3	0.2	2
Water transparency	m	0.3	0.1	0	0.6
Water temperature	°C	24	3	19	30
Ambient temperature	°C	26	2	20	31
Canopy cover	%	16	21	0	100

**Table 3:** Output of the logistic regression model to predict the occurrence of infected snails.

Variable	Estimate	Std. Error	z Value	Pr (>|z|)
Dissolved oxygen	−0.29322	0.11558	−2.537	0.0112 *
BOD_5_	0.011696	0.005558	2.104	0.0354 *

* Significant association (*p* < 0.05).

**Table 4:** Distribution of the infection intensities of *S. mansoni* among fishermen at Ethiopian Rift Valley lakes.

Study Area	Infection Intensity of *S. mansoni*, n (%)		
	Light	Moderate	Heavy
Lake Hawassa	22 (35)	19 (31)	21 (34)
Lake Ziway	17 (71)	4 (17)	3 (13)
Both lakes	39 (45)	23 (27)	24 (28)

n, the number of participants; % = percentage of participants categorized by the type of infection intensity.

**Table 5:** Multiple logistic regression analysis of factors associated with *S. mansoni* infection among the fishermen at Ethiopian Rift Valley lakes (n = number participants tested positive/negative for *S. mansoni*, % = percentage of participants tested positive/negative for *S. mansoni*).

Risk Factor	Category	*S. mansoni* Infection Status		COR (95% CI)	AOR (95% CI)
		Positive, n (%)	Negative, n (%)		
Age group (years)	18–27	19 (15)	104 (85)	0.40 (0.17–0.95) *	0.21 (0.07–0.64) *
	28–37	57 (24)	185 (76)	0.67 (0.31–1.46)	0.38 (0.13–0.96) *
	38 and above	11 (31)	24 (69)	1	1
Level of education	No formal education	11 (27)	30 (73)	1.1 (0.35–3.45)	1.77 (0.45–7.19)
	Primary education	70 (21)	265 (79)	0.79 (0.30–2.1)	0.97 (0.32–2.97)
	Secondary education and above	6 (25)	18 (75)	1	1
Residence	Urban	46 (22)	160 (78)	1.07 (0.67–1.74)	1.29 (0.73–2.27)
	Rural	41 (21)	153 (79)	1	1
Type of activity	Fishing	58 (27)	160 (73)	1.91 (1.16–3.15) *	2.24 (1.29–3.92) *
	Fish processing	29 (16))	153 (84)	1	1
Swimming/bathing in lake	Yes	75 (22)	264 (78)	1.16 (0.59–2.29)	1.08 (0.49–2.38)
	No	12 (20)	49 (80)	1	1
Open defecation/urination in lake	Yes	32 (32)	68 (68)	2.10 (1.26–3.50) *	2.37 (1.35–4.16) *
	No	55 (18)	245 (82)	1	1
Using water from lake for domestic purposes	Yes	13 (16)	70 (84)	0.61 (0.32–1.14)	0.33 (0.14–0.76) *
	No	74 (23)	243 (77)	1	1
Boiling water before drinking	Yes	3 (19)	13 (81)	0.84 (0.23–2.96)	0.67 (0.18–2.51)
	No	84 (22)	300 (78)	1	1
Defecating in bush	Yes	59 (21)	220 (79)	0.89 (0.53–1.49)	0.79 (0.42–1.48)
	No	28 (23)	93 (77)	1	1

Abbreviations: COR, crude odds ratio; AOR, adjusted odds ratio; CI confidence interval (an AOR has been adjusted to account for other predictor variables in a model). * Significant association (*p* < 0.05).

## Data Availability

The dataset generated and/or analyzed during the present study is available from the corresponding author.
